# *SLC19A3* Loss-of-Function Variant in Yorkshire Terriers With Leigh-Like Subacute Necrotizing Encephalopathy

**DOI:** 10.3390/genes11101215

**Published:** 2020-10-16

**Authors:** Michaela Drögemüller, Anna Letko, Kaspar Matiasek, Vidhya Jagannathan, Daniele Corlazzoli, Marco Rosati, Konrad Jurina, Susanne Medl, Thomas Gödde, Stefan Rupp, Andrea Fischer, Alejandro Luján Feliu-Pascual, Cord Drögemüller

**Affiliations:** 1 Institute of Genetics, Vetsuisse Faculty, University of Bern, 3012 Bern, Switzerland; michaela.droegemueller@vetsuisse.unibe.ch (M.D.); anna.letko@vetsuisse.unibe.ch (A.L.); vidhya.jagannathan@vetsuisse.unibe.ch (V.J.); 2 Section of Clinical & Comparative Neuropathology, Centre for Clinical Veterinary Medicine, Ludwig Maximilians Universität Munich, 80539 Munich, Germany; kaspar.matiasek@neuropathologie.de (K.M.); marco.rosati@neuropathologie.de (M.R.); 3 Neurology & Neurosurgery Unit, Policlinico Veterinario Roma Sud, 00173 Roma, Italy; daniele.corlazzoli@me.com; 4 Small Animal Hospital, Tierklinik Haar, 85540 Haar, Germany; datenschutz.haar@anicura.de; 5 Small Animal Hospital, Anicura Kleintierklinik Babenhausen, 87727 Babenhausen, Germany; smedl@tierklinik-medl.de; 6 Small Animal Referral Practice, Veterinary Health Centre, 83451 Piding, Germany; t.goedde@tierarzt-piding.com; 7 Small Animal Hospital, Tierklinik Hofheim, 65719 Hofheim, Germany; s.rupp@tierklinik-hofheim.de; 8 Section of Neurology, Centre for Clinical Veterinary Medicine, Ludwig-Maximilians-Universität, 80539 Munich, Germany; a.fischer@medizinische-kleintierklinik.de; 9 Aúna Especialidades Veterinarias, 46980 Valencia, Spain; alf@aunaespecialidadesveterinarias.es

**Keywords:** *Canis familiaris*, whole-genome sequencing, rare disease, precision medicine, neurometabolic disorder

## Abstract

Sporadic occurrence of juvenile-onset necrotizing encephalopathy (SNE) has been previously reported in Yorkshire terriers. However, so far, no causative genetic variant has been found for this breed-specific form of suspected mitochondrial encephalomyopathy. Affected dogs showed gait abnormalities, central visual defects, and/or seizures. Histopathological analysis revealed the presence of major characteristics of human Leigh syndrome and SNE in Alaskan huskies. The aim of this study was to characterize the genetic etiology of SNE-affected purebred Yorkshire terriers. After SNP genotyping and subsequent homozygosity mapping, we identified a single loss-of-function variant by whole-genome sequencing in the canine *SLC19A3* gene situated in a 1.7 Mb region of homozygosity on chromosome 25. All ten cases were homozygous carriers of a mutant allele, an indel variant in exon 2, that is predicted to lead to a frameshift and to truncate about 86% of the wild type coding sequence. This study reports a most likely pathogenic variant in *SLC19A3* causing a form of SNE in Yorkshire terriers and enables selection against this fatal neurodegenerative recessive disorder. This is the second report of a pathogenic alteration of the *SLC19A3* gene in dogs with SNE.

## 1. Introduction

Subacute necrotizing encephalomyelopathy (SNE), also termed Leigh syndrome (LS; OMIM 256000) represents a devastating neurodegenerative disorder in people, characterized by a wide variety of clinical signs, ranging from severe neurologic problems to a near absence of abnormalities with the central nervous system most frequently affected [1]. Originally, Archibald Denis Leigh, a British neuropsychiatrist described the condition in 1951 [2]. SNE is characterized by focal and bilaterally symmetrical, necrotic lesions involving the thalamus, brainstem, and posterior columns of the spinal cord [3]. In SNE, various mutations in mitochondrial respiratory chain complexes lead to the disruption of ATP synthesis resulting in the characteristic pathology of SNE [4]. Mitochondrial encephalomyelopathies, such as SNE or LS, represent rare inherited neurometabolic disorders showing considerable genetic heterogeneity and associated pathogenic variants affecting over 85 different genes of the mitochondrial or nuclear genome [3]. Therefore, they represent mitochondrial disorders with the largest genetic heterogeneity [1].

As human SNE is rare and heterogeneous, studying domestic animal species showing resembling conditions might add to the understanding of such a complex group of disorders. Rare forms of SNE were described e.g., in cattle [5,6,7] and dogs (OMIA 001097-9615). The first report of this disorder was described in Alaskan huskies [8,9] and subsequently, a similar form of SNE was reported in Yorkshire terriers [10] and American Staffordshire bull terriers [11]. Neuropathologically, SNE in Yorkshire terriers is nearly identical to the Alaskan husky form and very similar to human Leigh syndrome [10]. An initial genetic investigation of SNE-affected Yorkshire terriers revealed no indication for disease-causing variants in the mitochondrial genome [10], whereas more recently in Alaskan huskies the pathogenesis of recessively inherited SNE was unraveled [12,13]. This breed-specific fatal brain disorder in Alaskan huskies is associated with a deleterious loss-of-function variant in *SLC19A3* encoding for a thiamine transporter 2 (THTR2) with a predominately central nervous system (CNS) distribution [12,13]. The *SLC19A3* gene product controls the uptake of thiamine in the CNS via expression of the thiamine transporter protein THTR2. Pathogenic variants are associated with thiamine metabolism dysfunction syndrome-2 in people (THMD2; OMIM 607483), also known as biotin-responsive basal ganglia disease (BBGD) or thiamine-responsive encephalopathy [14]. This *SLC19A3*–related condition is an autosomal recessive disorder with childhood-onset that presents as a subacute encephalopathy and progresses to severe cogwheel rigidity, dystonia, quadriparesis, and eventually death if left untreated (OMIM 606152). The *SLC19A3*–related SNE of Alaskan huskies was proposed as a possible large animal model that may allow prospective investigations into the mechanisms of *SLC19A3*-related syndromes and the potential role of thiamine and/or biotin as a therapeutic strategy [12,13].

To elucidate the disease mechanism underlying monogenic autosomal recessive inherited SNE in Yorkshire terriers, we applied homozygosity mapping and whole-genome sequencing revealing a most likely pathogenic variant in the canine *SLC19A3* gene.

## 2. Materials and Methods

### 2.1. Ethics Statement

All animal experiments were performed according to the local regulations. All animals in this study were examined with the consent of their owners. Sample collection was approved by the Cantonal Committee for Animal Experiments (Canton of Bern; permit 71/19).

### 2.2. Animals

In total, 172 blood samples of Yorkshire terriers were collected. Ten dogs were diagnosed with Leigh-like subacute necrotizing encephalopathy (SNE) according to Baiker et al. [10]. These affected dogs were unrelated, apart from two full siblings for which their sire and dam (obligate carriers) as well as a single normal littermate were also available. The remaining 159 dogs represented unrelated purebred controls. Genomic DNA was isolated from EDTA blood samples using the Maxwell RSC Whole Blood DNA kit (Promega, Dübendorf, Switzerland).

### 2.3. Single Nucleotide Polymorphism Array Genotyping

Four selected SNE-affected Yorkshire terriers were genotyped on Illumina CanineHD BeadChip array (Illumina, San Diego, CA, USA). PLINK v1.9 [15] was used to perform the quality control filtering steps of the obtained genotyping data and the subsequent homozygosity analysis. Single nucleotide polymorphisms (SNP) with a call rate <90% were removed leaving 167,185 markers. All individuals had call rates >90%. Homozygosity analysis was carried out with PLINK v1.9 [15] to determine intervals of extended homozygous regions with alleles shared by all four affected dogs.

### 2.4. Whole-Genome Sequencing

Whole-genome sequence (WGS) data of a single affected dog was obtained at 19.7× coverage in order to identify the causative variant for SNE. The sequence data analysis and calling of single nucleotide variants and small indels (SNVs) including the prediction of functional effects were described before [16]. The dog reference genome assembly CanFam3.1 and NCBI annotation release 105 was used. Additionally, a publicly available control genomes cohort of 720 dogs from 130 various breeds, and 9 wolves [16] was used to filter variants private in the sequenced SNE-affected dog; this also included 60 unrelated Yorkshire terriers (Supplementary Table S1). The Integrative genomics viewer (IGV) software [17] was used for visual inspection and screening for structural variants in the associated regions.

### 2.5. Sanger Sequencing and Targeted Genotyping

Polymerase chain reaction (PCR) and Sanger sequencing were used to validate and characterize the *SLC19A3* indel variant (XM_022409850.1:c.205_210delins35) identified from whole-genome sequencing. PCR primers were designed using primer 3 [18]. PCR products from genomic DNA were amplified using AmpliTaqGold360 MasterMix (Thermo Fisher Scientific, Waltham, MA, USA) and the purified PCR amplicons were directly sequenced on an ABI3730 capillary sequencer (Thermo Fisher Scientific) using the following primers: GGCAGTCACCATCCCATAGA (forward) and GATATTGGGCAAGCCACCTA (reverse) generating 309 bp products. The sequence data were analyzed with Sequencher 5.1 software (GeneCodes, Ann Arbor, MI, USA). Diagnostic genotyping was performed by fragment length analysis using a different forward primer (ATCCCTTGCAGGATGATGAC) to produce amplicons of 218 bp or 247 bp representing the wild type or variant allele, respectively. The 29 bp size difference was visualized on a Fragment Analyzer capillary gel electrophoresis instrument (Advanced Analytical Technologies, Ames, IA, USA).

### 2.6. Availability of Data and Material

The whole-genome data of an SNE-affected Yorkshire terrier are freely available at the European Nucleotide Archive (ENA) under sample accession number SAMEA3928145. All accession numbers of the used control genomes are available in Supplementary Table S1.

All genome positions are reported with respect to dog reference genome assembly CanFam3.1 and NCBI annotation release 105. All references to the canine *SLC19A3* gene correspond to the accessions NC_006607.3 (NCBI accession), XM_022409850.1 (mRNA), and XP_022265558.1 (protein).

## 3. Results

### 3.1. Homozygosity Analysis

Based on the clinicopathological diagnosis of Leigh-like subacute necrotizing encephalopathy (SNE) in all examined Yorkshire terriers and the similarities to the recessively inherited conditions in SNE-affected Alaskan husky dogs and THMD2-affected humans, as well as the available pedigree information of the two SNE-affected siblings, a recessive mode of inheritance was postulated. Therefore, homozygosity mapping assuming identity-by-descent (IBD) was used to determine critical genomic regions shared across four SNP array genotyped cases. This revealed five genome regions with a total of ~4.1 Mb located on five different dog chromosomes (Table 1), representing 0.17% of the canine reference sequence. Visual inspection of these regions in the WGS of the affected dog did not reveal any evidence for copy number variants or large structural rearrangements.

### 3.2. Identification of the Causative Variant

**Figure 1:**
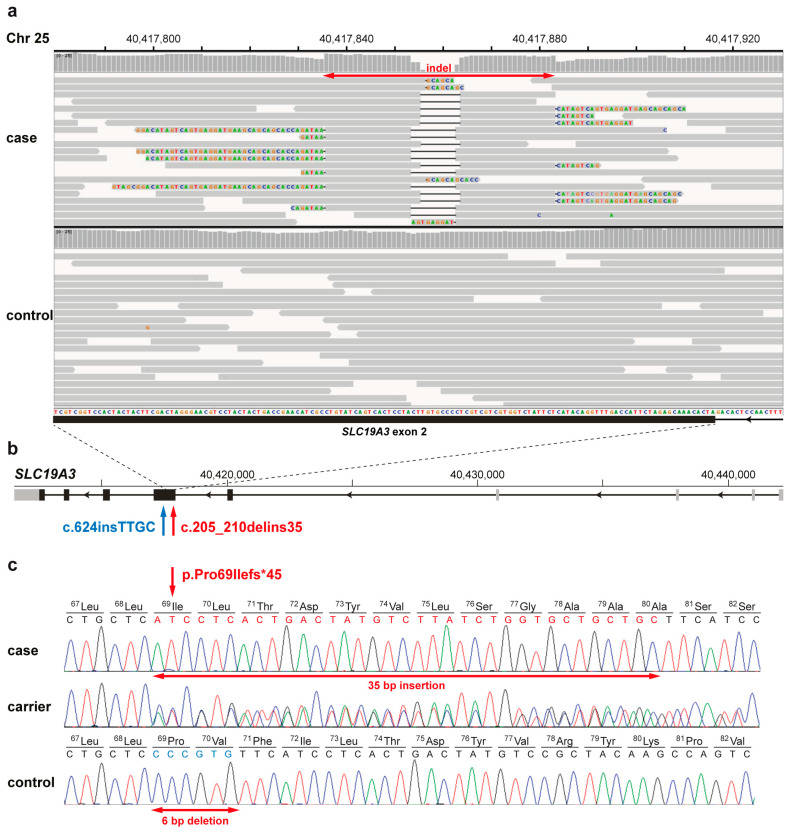
Subacute necrotizing encephalopathy (SNE)-associated *SLC19A3* loss-of-function variant in Yorkshire terriers. (**a**) IGV [17] screenshots of the genome region on canine chromosome 25 with the *SLC19A3*:c.205_210delins35 variant in an affected and a control Yorkshire terrier (NC_006607.3:40417780-40417930); The indel variant detected in the SNE-affected dog is indicated by a red arrow. (**b**) Schematic representation of the canine *SLC19A3* gene showing the location of both pathogenic variants in exon 2 (XM_022409850.1): the herein identified indel (red arrow) and the insertion previously described in encephalopathy-affected Alaskan huskies (blue arrow) [12]. Note that the number of 5′-untranslated exons (grey) varies between transcript isoforms, whereas the five protein-coding 3′-exons (black) are more conserved; (**c**) Sanger sequencing electropherograms illustrate sequences of a homozygous SNE-affected Yorkshire terrier, a heterozygous carrier, and a homozygous wild type dog. The red arrows indicate that the 35 bases shown in red are inserted, whereas the 6 bases in blue are deleted in the mutant allele. The predicted consequence of the shift in the reading frame altering the amino acid sequence of the SLC19A3 protein and leading to a premature stop is shown above.

Filtering the variants of a single affected Yorkshire terrier against 729 public control genomes [16], including 60 breed controls, for single-nucleotide variants (SNVs) and short indels present in the five identified IBD-regions resulted in only a single private protein-changing variant (Figure 1a). The indel affecting ~45 bp is located in exon 2 of the *thiamine transporter 2* (*LOC486151*) gene, also known as solute carrier family 19 members 3 (*SLC19A3*) gene (Figure 1b). PCR and subsequent Sanger sequencing confirmed the homozygous presence of this small structural variant in SNE-affected Yorkshire terriers and revealed the detailed features of the indel: a 35 bp insertion replacing 6 bp and thereby disturbing the correct reading frame (Figure 1c). There are 15 currently annotated transcript isoforms for the canine *SLC19A3*, which is in reverse complementary orientation with respect to the canine reference genome. While the canine SLC19A3 protein length is 495 amino acids, the human protein (NP_001358340.1) has 496 amino acids, from which 408 (82.3%) are identical between dog and human. The Yorkshire terrier variant leads to a frameshift and a premature stop codon (c.205_210delins35; p.Pro69Ilefs*45) truncating ~86% of the wild type coding sequence (Figure 1c).

### 3.3. Targeted Genotyping of the Variant

Genotyping by fragment size analysis of the 172 available Yorkshire terriers confirmed perfect segregation of the detected *SLC19A3* variant with the observed disease phenotype. Only the ten SNE-affected dogs were homozygous for the variant allele (Table 2). Two obligate carriers and one tested normal littermate were heterozygous carriers of the variant, while 162 controls tested homozygous for the wild type allele (Table 2).

## 4. Discussion

In this study, the obtained genetic results elucidate the underlying aetiology of the previous clinical and pathological characterization of a Leigh-like subacute necrotizing encephalopathy in the affected Yorkshire terriers, which resembles the human Leigh syndrome. The *SLC19A3* variant found by a combination of SNP genotyping-based homozygosity mapping and whole-genome sequencing, confirmed by Sanger sequencing, segregated perfectly in the investigated cohort of >200 unrelated Yorkshire terriers.

Numerous homozygous as well as compound heterozygous variants have been reported before in different regions of *SLC19A3* in human patients suffering from thiamine metabolism dysfunction syndrome-2 [19]. *SLC19A3* is a member of solute carrier family 19 and encodes thiamine transporter 2. Together with thiamine transporter 1, it is necessary for transport and homeostasis of thiamine that is important in brain development. [20]. *Slc19a3*-knockout mice showed progressive wasting and lethargy leading to a premature death as well as a significant decrease in thiamin uptake, even though there were no obvious histological changes in the brain [21].

The herein-described most likely pathogenic variant (XP_022265558.1:p.Pro69Ilefs*45) lies within the second of 12 transmembrane domains of the SLC19A3 protein and, therefore, affects ~86% of the wild type sequence. The *SLC19A3* gene probability of loss-of-function intolerance is pLI = 0.104 [22], which indicates variants in *SLC19A3* leading to a loss of gene function are most likely recessive, where loss of a single copy is often tolerated but the loss of both copies is not. The herein-described variant leads to an insertion of a premature termination in the second out of five coding exons, suggesting that any synthesized mRNA would likely be degraded through nonsense-mediated decay, unlikely to produce a fully functional protein. Heterozygous carriers did not show a visible clinical phenotype, as they can most likely compensate due to the presence of the normal protein, albeit at a decreased amount.

In conclusion, our results provide strong evidence for a breed-specific deleterious variant in *SLC19A3* as the most likely genetic cause of monogenic autosomal recessive Leigh-like subacute necrotizing encephalopathy in Yorkshire terriers, and they enable the development of a genetic test for veterinary diagnostic and breeding decisions. Finally, this presents the second, most likely breed-specific pathogenic variant in the canine *SLC19A3* gene in SNE-affected dogs.

## Figures and Tables

**Table 1:** Regions of shared homozygosity detected in four subacute necrotizing encephalomyelopathy (SNE)-affected Yorkshire terriers.

Chromosome	Position ^1^		Length (kb)	Number of Annotated Protein-Coding Genes in the Region
	Start	End		
3	44,184,889	44,286,148	101.3	0
6	71,329,720	71,552,171	222.5	1
10	20,608,121	22,376,735	1768.6	22
25	39,477,619	41,191,570	1714.0	13
31	33,337,422	33,591,249	253.8	2

^1^ in respect to dog reference genome assembly CanFam3.1.

**Table 2:** Segregation of the *SLC19A3*: c.205_210delins35 genotypes with subacute necrotizing encephalopathy in Yorkshire terriers.

SNE Status	wt/wt	wt/var	var/var
Affected (*n* = 10)	0	0	10
Non-affected (*n* = 222) ^1^	219	3 ^2^	0

^1^ including 60 dogs with WGS data ^2^ includes 2 obligate carriers and 1 normal littermate of the affected dogs.

## Supplementary Materials

The following are available online at https://www.mdpi.com/2073-4425/11/10/1215/s1, Table S1: Accession numbers of 721 dog and 9 wolf whole-genome genome sequences.
